# The American Liveryman and Horse-owner

**Published:** 1884-10

**Authors:** 


THE AMERICAN LIVERYMAN AND HORSE-OWNER.

This is the title of a periodical the publication of which has just been commenced by J. Jackson, Esq., Chicago. It will make its appearance monthly, and the price is $1.00, or 50 cents if subscribed for before October 1st. Much information is to be found in it valuable to all those who take interest in livery matters.

